# A high-quality reference genome of wild *Cannabis sativa*

**DOI:** 10.1038/s41438-020-0295-3

**Published:** 2020-05-02

**Authors:** Shan Gao, Baishi Wang, Shanshan Xie, Xiaoyu Xu, Jin Zhang, Li Pei, Yongyi Yu, Weifei Yang, Ying Zhang

**Affiliations:** 1 Institute of Forensic Science, Ministry of Public Security, No. 17 South Muxidi Lane, Xicheng District, Beijing, 100038 China; 2 Beijing Century Legend Bioscience Co., Ltd., Beijing, 102300 China

**Keywords:** Plant breeding, Plant evolution, Structural variation

## Abstract

*Cannabis sativa* is a well-known plant species that has great economic and ecological significance. An incomplete genome of cloned *C. sativa* was obtained by using SOAPdenovo software in 2011. To further explore the utilization of this plant resource, we generated an updated draft genome sequence for wild-type varieties of *C. sativa* in China using PacBio single-molecule sequencing and Hi-C technology. Our assembled genome is approximately 808 Mb, with scaffold and contig N50 sizes of 83.00 Mb and 513.57 kb, respectively. Repetitive elements account for 74.75% of the genome. A total of 38,828 protein-coding genes were annotated, 98.20% of which were functionally annotated. We provide the first comprehensive *de novo* genome of wild-type varieties of *C. sativa* distributed in Tibet, China. Due to long-term growth in the wild environment, these varieties exhibit higher heterozygosity and contain more genetic information. This genetic resource is of great value for future investigations of cannabinoid metabolic pathways and will aid in promoting the commercial production of *C. sativa* and the effective utilization of cannabinoids. The assembled genome is also a valuable resource for intensively and effectively investigating the *C. sativa* genome further in the future.

## Introduction

*Cannabis sativa* L., a native plant of Central Asia, is first cultivated in Asia and Europe and is now one of the most popularly cultivated plants worldwide^1^. In China, hemp fiber has been used to produce textiles for the past 6000 years^2^.

*C. sativa* is one of the most valuable agriculturally important crops in nature. Although it is widely used to produce paper, textiles, building materials, food, and medicine, a secondary metabolite, tetrahydrocannabinol (THC), is also used to produce well-known drugs. Frequent, long-term, selective breeding has produced both hemp fiber and medicinal cannabis strains, with medicinal cannabis showing promise in effectively treating various diseases^3^ by relieving an array of symptoms, including pain, nausea, anxiety, and inflammation^4–7^. The therapeutic efficacy of medicinal cannabis is mainly dependent on cannabinoids, which are endemic metabolites unique to *C. sativa*^8^, among which THC and cannabidiol (CBD) are the main chemical cannabinoid compounds.

Although cannabis has considerable economic and medical value, information about its genome is limited. While a genomic draft was published recently, in 2011^9^, the splicing of this draft was neither of good quality nor complete, thus hindering its usefulness.

Cannabis is mostly dioecious, with a diploid genome (2*n* = 20) containing nine pairs of autosomes and one pair of sex chromosomes (female plants (XX) and male plants (XY)). The Y chromosome is larger than the X chromosome, and the female plant’s haploid genome is estimated to be 818 Mb in size, while the male plant’s genome is estimated to be 843 Mb^10,11^. However, the published genome is for cloned *C. sativa* and was assembled using SOAPdenovo software to obtain a genome of approximately 786 Mb^9^. It shows a contig N50 = 2.8 kb and scaffold N50 = 16.2 kb, and genome annotations are missing. Additionally, the genome assembly quality is poor since it contains incomplete assembly of gene regions and repeat sequences.

The cannabis genome has been sequenced^9^, but the sequenced plant came from a cultivated variety. Generally, cultivated varieties lose substantial genetic diversity through successive bottlenecks due to domestication and selection for traits to increase yield under intensive human cultivation^12^. Therefore, wild-type varieties are an important source of genetic diversity for molecular breeding. In this report, we performed genomic sequencing, assembly, annotation, and evolutionary analysis in wild-type varieties of *C. sativa*. The genetic data obtained in this study will be a valuable resource for future studies assessing the pharmacology, chemical constituents, cultivation, and genetic improvement of the traits of these plants and could be used as a reference in future population genetic studies of *C. sativa*.

## Results

### Sample collection and sequencing

One female of the wild-type *C. sativa* “JL” variety was used for whole-genome sequencing in this study. The sequencing depth was 153×, and 124 Gb of genomic data were obtained. The subread N50 was 13.5 kb (Table 1). Additionally, we performed next-generation, paired-end sequencing and finally obtained a total of 95.97 Gb of clean data. These data were then used to evaluate the quality of the wild-type *C. sativa* “JL” variety genome.

**Table 1:** Statistical results for PacBio sequencing data

Sample	Cells	Subreads_reads (bp)	Subreads_base (bp)	Average_subreads_length (bp)	Accuracy	Subreads_n50 (bp)	GC_mean
JL	12	12,944,138	124,211,451,985	9,595.96	0.8	13,485	0.38

RNA sequencing data were used for genome function annotation. Using paired-end sequencing, we obtained 42.1 Gb of clean data, with each sample producing 6.5–9.8 Gb (Supplementary Information; Table S1).

### Genome assembly

A variety of methods were used for genome assembly, and the initial assembly yielded a genome size of 811,814,330 bp, with a contig N50 of 632,748 bp. After assembling the third-generation subreads, the next-generation data were used to correct the genome map. For this purpose, we used BWA (v0.7.9a, RRID: SCR 010910) to compare the next-generation clean reads with the assembled sequence, and based on the comparison results, we corrected the sequence using Pilon (v1.22, Broad Institute, MA, USA)^13^. The post-correction genome size was 812,295,151 bp, with a contig N50 of 633,146 bp. Statistical analysis of base pairs in the corrected genome showed that the average GC content in the genome was 33.8%. The contents of other base pairs are presented in Supplementary Information; Table S2.

### Hi-C

The Hi-C approach efficiently uses high-throughput sequencing to determine the state of genome folding by measuring the frequency of contact between pairs of loci^14,15^. Originally, this technique was developed to generate chromosomal genome assemblies, but it was subsequently found to be useful for genome-wide chromosome conformation capture^16^.

Nearly 487 million raw reads (146.25 Gb) were collected and then reduced to 424 million clean reads after filtering out low-quality reads and retaining reads with more than 5% N bases, adapter reads, and single reads. We then successfully clustered, ordered and oriented 2,506 contigs into 10 groups according to the agglomerative hierarchical clustering method in Lachesis (https://github.com/shendurelab/LACHESIS)^17^, representing 91% and 99% of the total genome by contig number and base count, respectively (Table 2). Along with Hi-C analysis, we visually inspected contig orientation and suspicious fragments and found little incorrect information, and we identified and corrected that information using self-written scripts. We obtained a high-quality chromosome-level cannabis genome with a contig N50 of 513 kb and a scaffold N50 of 83 Mb (Table 3). According to a heatmap of the contig contact matrix with Hi-C data (Supplementary Information; Fig. S1), we estimated that the clustering, ordering, and orientation of the contigs was valid (Table 4). Among these contigs, the scaffold N50 was 162 times greater than the scaffold N50 of the preliminarily assembled genes. There were 2,506 mounted and 245 unmounted scaffolds on the chromosomes (Table 5). Our *C. sativa* genome is a solidly based genomic resource for cultivar identification, population analysis, and functional analysis.

**Table 2:** Summary of contig/scaffold clustering results

Sample	JL
Number of sequences in draft genome	2,752
Length of sequences in draft genome (bp)	807,650,591
Number of sequences in clustering	2,506
Rate of numbers in clustering (%)	91.06
Length of sequences in clustering (bp)	797,989,137
Rate of numbers in clustering (%)	98.80

**Table 3:** Summary of Hi-C auxiliary assembly results

Items	Contig_len (bp)	Contig_num	Scaffold_len (bp)	Scaffold_num
Total	807,650,591	2,752	807,900,192	255
Max	2,865,895	–	93,001,284	–
Number ≥ 2000 bp	–	2,751	–	255
N50	513,574	464	82,998,198	5
N60	401,967	641	82,468,740	6
N70	301,581	873	80,615,893	7
N80	214,974	1,189	70,972,478	8
N90	130,882	1,661	69,092,163	9

**Table 4:** Contig/scaffold sorting results

Sample	JL
Number of sequences in ordering	2,506
Rate of numbers in ordering (%)	99.88
Length of sequences in ordering	797,739,537
Rate of lengths in ordering (%)	99.99
Number of sequences in trunks	700
Rate of numbers in trunks (%)	27.93
Length of sequences in trunks	405,630,183
Rate of lengths in trunks (%)	50.85

**Table 5:** Summary of Hi-C-assisted assembly pseudomolecule lengths

Pseudomolecule	Scaffold Num	Length
Chr1	297	93,001,284
Chr2	241	91,276,498
Chr3	314	89,817,320
Chr4	260	83,221,442
Chr5	258	82,998,198
Chr6	297	82,468,740
Chr7	235	80,615,893
Chr8	209	70,972,478
Chr9	264	69,092,163
Chr10	131	54,525,121
Total anchored	2,506	797,989,137
Unanchored	245	9,911,055

### Assessment of genomic integrity

BUSCO (v3.0)^18^ was employed to evaluate the accuracy and completeness of our genome assembly, gene set, and transcripts. Based on the OrthoDB (http://cegg.unige.ch/orthodb) database, BUSCO built several large, single-copy gene sets covering the branches of the evolutionary tree. When comparing the gene set to the genome, it was noted that the proportion of complete BUSCOs was 92.6% (Supplementary Information; Table S3), indicating that the genome assembly integrity was very good.

Due to potential contamination during sequencing and assembly, we further evaluated our genome assembly by using GC depth analysis. The GC depth scatter plot showed no significant differentiation, and points were concentrated around the 34% area, indicating high assembly quality without any bacterial contamination (Supplementary Information; Fig. S2). Finally, the sequencing profile base depth was close to a Poisson distribution, further indicating that the assembled genome showed high assembly quality (Supplementary Information; Fig. S3).

To evaluate the consistency of the next-generation data, we compared the sequencing reads to the assembled scaffold sequences, and the resultant comparison ratio for the reads and genomic coverage showed that they were deep and complete (Supplementary Information; Fig. S4). The comparison rate of the next-generation data was 96.77% (Supplementary Information; Table S4), indicating that the assembled genome was of high quality.

### Genome annotation

Repeat sequences, including tandem repeats and interspersed repeats, are important components in the genome, and there are two strategies for predicting such sequences. The total lengths of transposon elements obtained from the genome sequence were 118,700,582 and 161,847,743 bp, representing 14.61% and 19.92% of the genome, respectively. For *de novo* prediction, RepeatModeler (v1.0.8) was used to first establish a *de novo* repeat sequence library, after which the genome sequence was predicted using RepeatMasker (v4.0.6). These results revealed a total length of 584,319,477 bp, representing 71.93% of the genome. In addition, we used the *de novo* prediction method with Tandem Repeat Finder (v4.09, https://tandem.bu.edu/trf/trf.html) to identify tandem repeats in the genome. The total length obtained by this method was 22,382,718 bp, representing 2.76% of the genome. Finally, we removed overlapping portions of the non-redundant repeats that we identified with those procedures, resulting in 612,733,451 bp of non-redundant repeats, which accounted for 74.75% of the assembled genome (Supplementary Information; Table S5; Figshare 1). Long terminal repeats (LTRs) represented 50% of the repeat sequences in the assembled genome (Supplementary Information; Table S6).

**Fig. 1:**
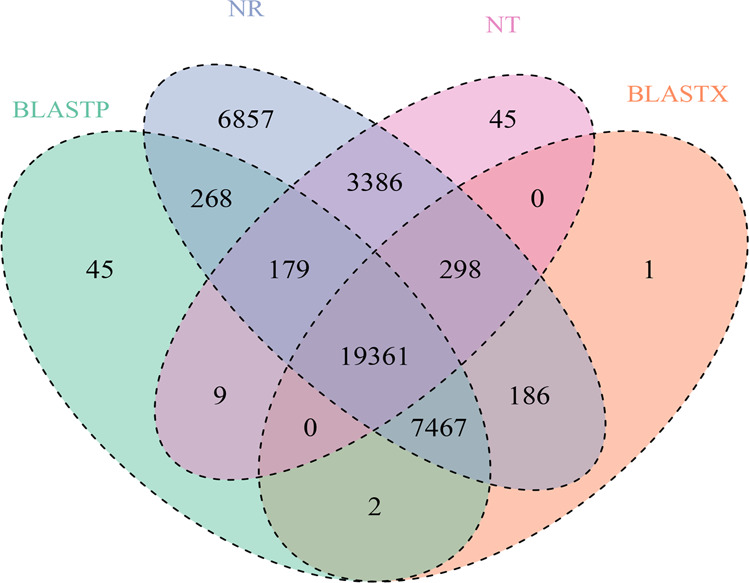
Gene annotation Venn diagram. Comparison of the annotated gene results from the NT, NR, UniProt-BLASTX, and UniProt-BLASTP databases

After predicting the gene approval rating, we used CPC (v0.9-r2)^19^ to analyze the potential of the predicted genes and to generate 38,828 predicted genes. The average length of the predicted genes was 551.10 bp, and the average length of their coding sequences was 1153.47 bp. There were an average of four exons per gene with a length of 281.91 bp per exon, and the average intron length was 2147.77 bp. After comparing our predicted gene set with the functional databases SwissProt, NT (https://www.ncbi.nlm.nih.gov/nucleotide/), NR, PFAM^20^, eggNOG (http://eggnogdb.embl.de/)^21^, GO (http://geneontology.org/page/go-database)^22^, and KEGG^23^, 38,129 genes were annotated, accounting for 98.20% of the genome (Table 6, Figshare 2). These results were statistically graphed in the NT, NR, UniProt-BLASTX, and UniProt-BLASTP databases (Fig. 1).

**Table 6:** Summary of gene function annotations

Database	Count	Percentage (%)
BLASTP	27,331	70.39
BLASTX	27,315	70.35
GO	27,931	71.94
KO	8,720	22.46
Map	5,008	12.90
NR	38,002	97.87
NT	23,278	59.95
PFAM	29,633	76.32
eggNOG	19,286	49.67
Total_anno	38,129	98.20
Total_unigene	38,828	100

We predicted non-coding RNAs, such as rRNAs, tRNAs, snRNAs, and miRNAs, by comparing their sequences with the known non-coding RNA library Rfam^24^. A total of 2,441 rRNAs, 214 snRNAs, and 281 miRNAs were thus predicted (Supplementary Information; Table S7, Figshare 3, 4, 5). tRNAscan-SE^25^ was used to predict tRNA sequences in the genome, resulting in 712 tRNAs (Supplementary Information; Table S7). To further verify our gene annotation results, we conducted a BUSCO evaluation using the embryophyta_odb10 database, producing a result of 93%, indicating that the annotation results were acceptable (Supplementary Information; Table S8).

### Gene families and phylogenetic analysis

**Fig. 2:**
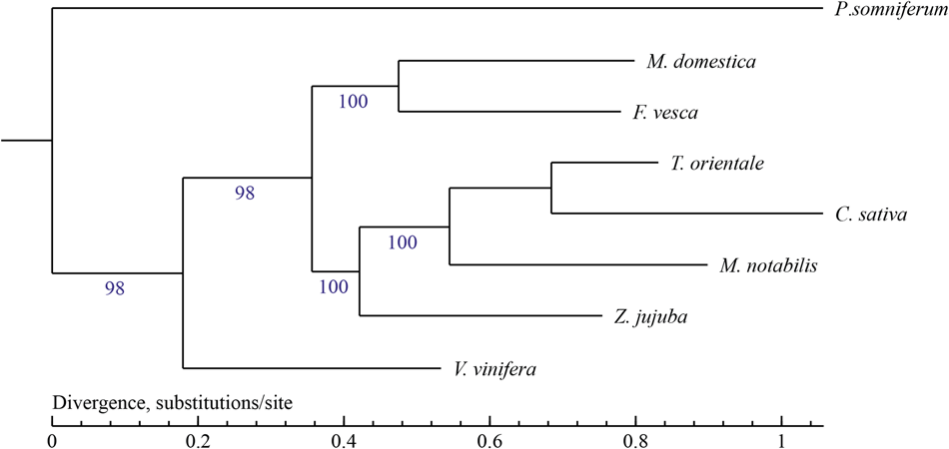
*C. sativa* phylogenetic tree. The longer the branch length, the longer the divergence time. The closer the branches, the closer the predicted genetic relationship. In general, we considered a bootstrapping value above 85 to represent good support for the result. Numbers above the nodes are bootstrap values

OrthoMCL (v1.4)^26^ was used to classify gene families with single and multiple copies from both closely related and remotely related species. (Supplementary Information; Table S9 and Fig. S5), resulting in the identification of 930 *C. sativa*-specific genes. *C. sativa* shows more genes in common with *Trema orientale* and *Morus notabilis* than with other species (Supplementary Information; Fig. S6). We used MUSCLE software (v3.8.31)^27^ to perform multiple sequence alignments for all single-copy gene families sequences. After we constructed the integrated supergene sequence, which was based on the four-fold degenerated sites (4DTv sites) of orthologous family genes, we used PhyML (v3.0)^28^ to construct the species phylogenetic tree (ML-Tree). As shown in Fig. 2, *Vitis vinifera* and *Fragaria vesca* in Rosaceae diverged from one another earlier than *T. orientale*, *M. notabilis*, and *Ziziphus jujuba* diverged from each other, and *C. sativa* is most closely related to *T. orientale*.

**Fig. 3:**
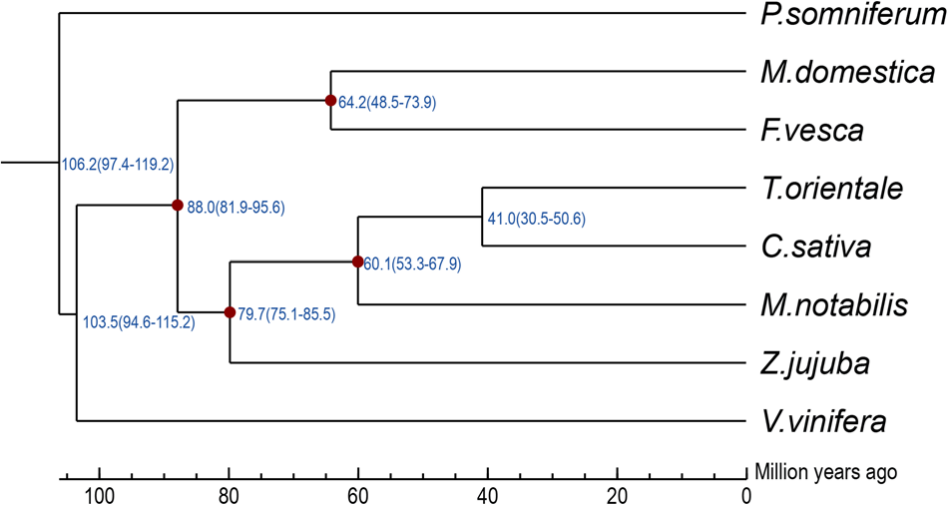
Phylogenetic relationships and divergence time. The blue numbers at the node positions represent the divergence time of each species in millions of years (Ma). The numbers in parentheses indicate the confidence interval of the divergence time, which can be used to estimate the divergence time of target species and other species. The red dots are the calibration time used to correct the time of species divergence

Based on our phylogenetic analysis of the integrated supergene sequence, we used PAML’s MCMCTREE software (v4.4)^29^ and the Bayesian relaxed molecular clock method to estimate divergence time. We used corrected time directly obtained from TimeTree^30–33^ (see Supplementary Material 10). The divergence time corresponding to the crown clade of Eudicots was 115 Ma (Fig. 3).

### Whole-genome duplication and gene families expansion/contraction analysis

**Fig. 4:**
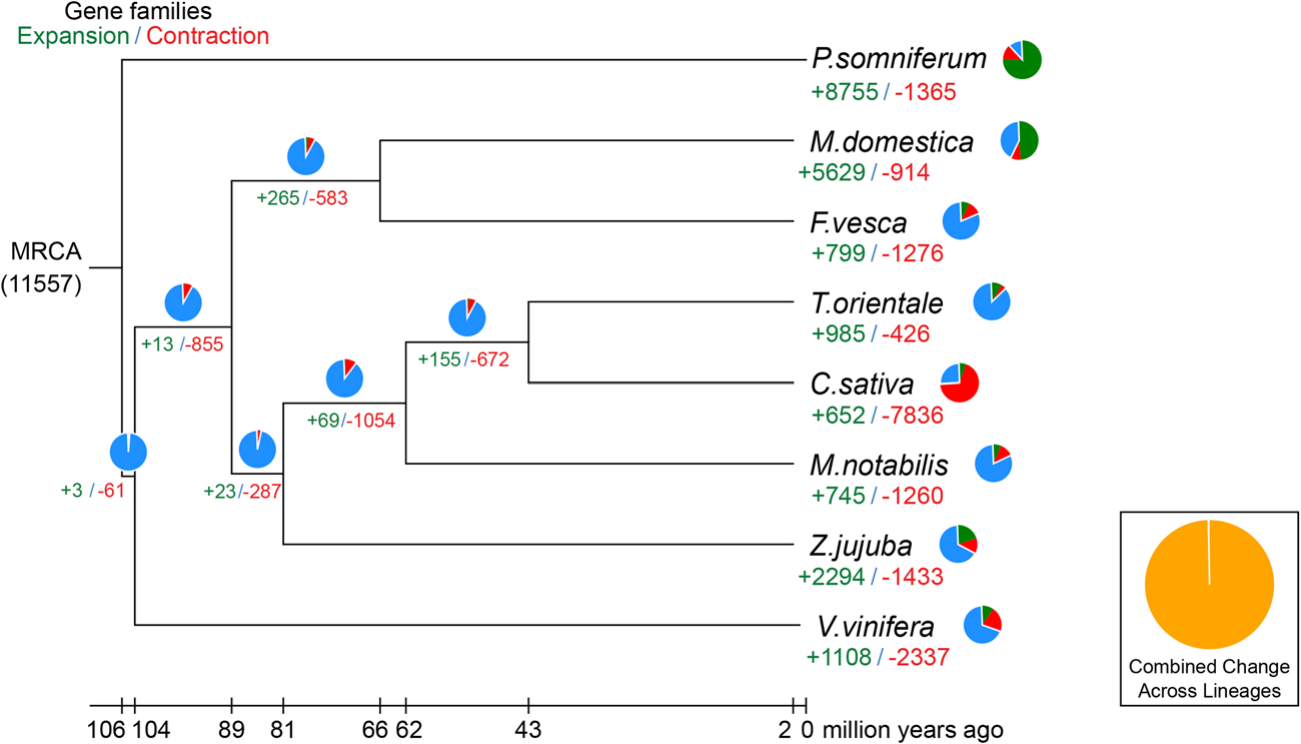
Fourfold degenerate site (4DTv) value distribution. Green numbers represent the number of gene families present when a species expanded during evolution, and red numbers represent the number of gene families present when a species contracted during evolution

Gene families expansion and contraction were analyzed based on mathematical statistical tests. After the cluster analysis of gene families, those with abnormal gene numbers in individual species were filtered, and CAFE (v4.1)^34^ and probabilistic graphical models (PGMs) were then used to simulate the acquisition and loss of genes under the specified phylogenetic tree and to analyze gene families expansion and contraction using hypothesis testing (Fig. 4). We found 12,801 gene families in the MCRA (most recent common ancestor). In comparison to *M. notabilis*, *T. orientale, V. vinifera, F. vesca*, *Musca domestica*, *Z. jujuba*, and *Papaver somniferum*, there were 2,599 gene families showing expansion and 1,298 gene families showing contraction in *C. sativa*.

**Fig. 5:**
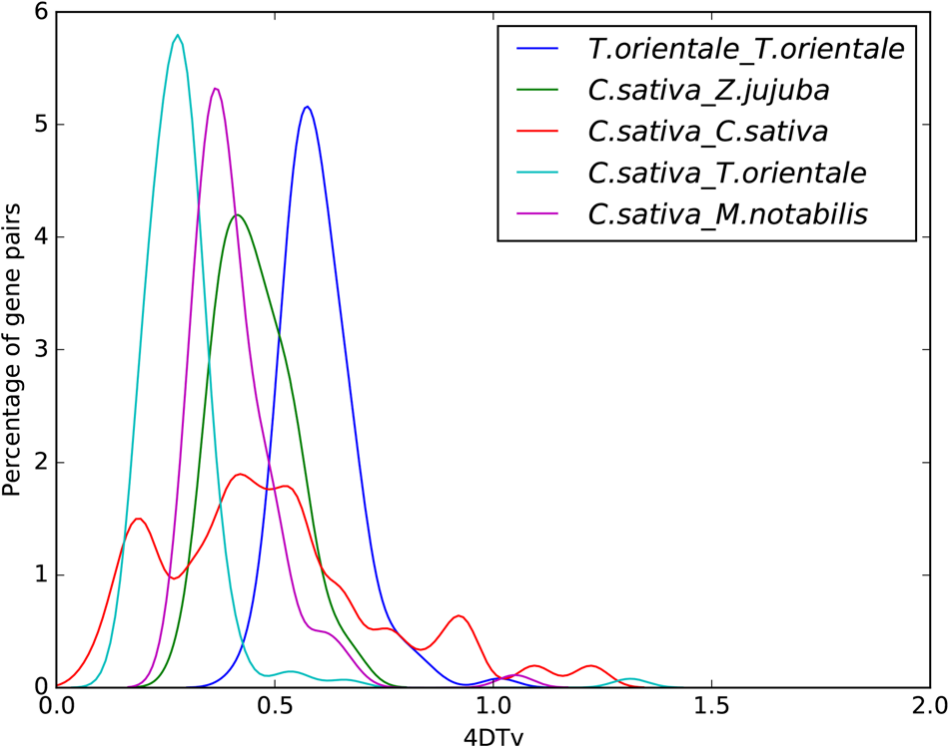
4DTv. The abscissa represents the 4DTv value, and the ordinate represents the proportion of genes corresponding to the 4DTv values

Whole-genome duplication events are widespread in plants and are a potent force that drives plant genome evolution. We used MCscan (v.0.8)^35,36^ to identify genome synteny blocks within *C. sativa* and other related species. Using MUSCLE (v3.8.31)^27^, we performed multiple sequence alignment of the internal sequences of the blocks and then calculated 4DTv site values. Based on the abundance of 4DTv site values, we estimated the relative timing of whole-genome duplication (WGD) or species split events. In general, three significant peaks were seen in the *C. sativa* genome (4DTv ~0.19, ~0,42, and ~0.92; Fig. 5), suggesting that *C. sativa* has experienced three WGD events. We also identified the 4DTv values from collinear blocks between *C. sativa* and the genomes of three closely related species, *M. notabilis*, *T. orientale*, and *Z. jujuba*. Additionally, we identified two ancient WGD events in *T. orientale* with two peaks at ~0.58 and 1.0. The γ event occurred after the divergence between monocots and dicots approximately 185 ± 55 million years ago (Mya)^37^. Therefore, this pattern indicates that *C. sativa* experienced large-scale gene duplication more recently than *T. orientale*, 35 Mya. The divergence of *C. sativa* and *T. orientale* occurred at ~52 Mya (4DTv ~0.28), and *C. sativa* and *T. orientale* shared two WGD events (4DTv ~0.42 to ~0.58 and ~0.92 to ~1.0).

**Fig. 6:**
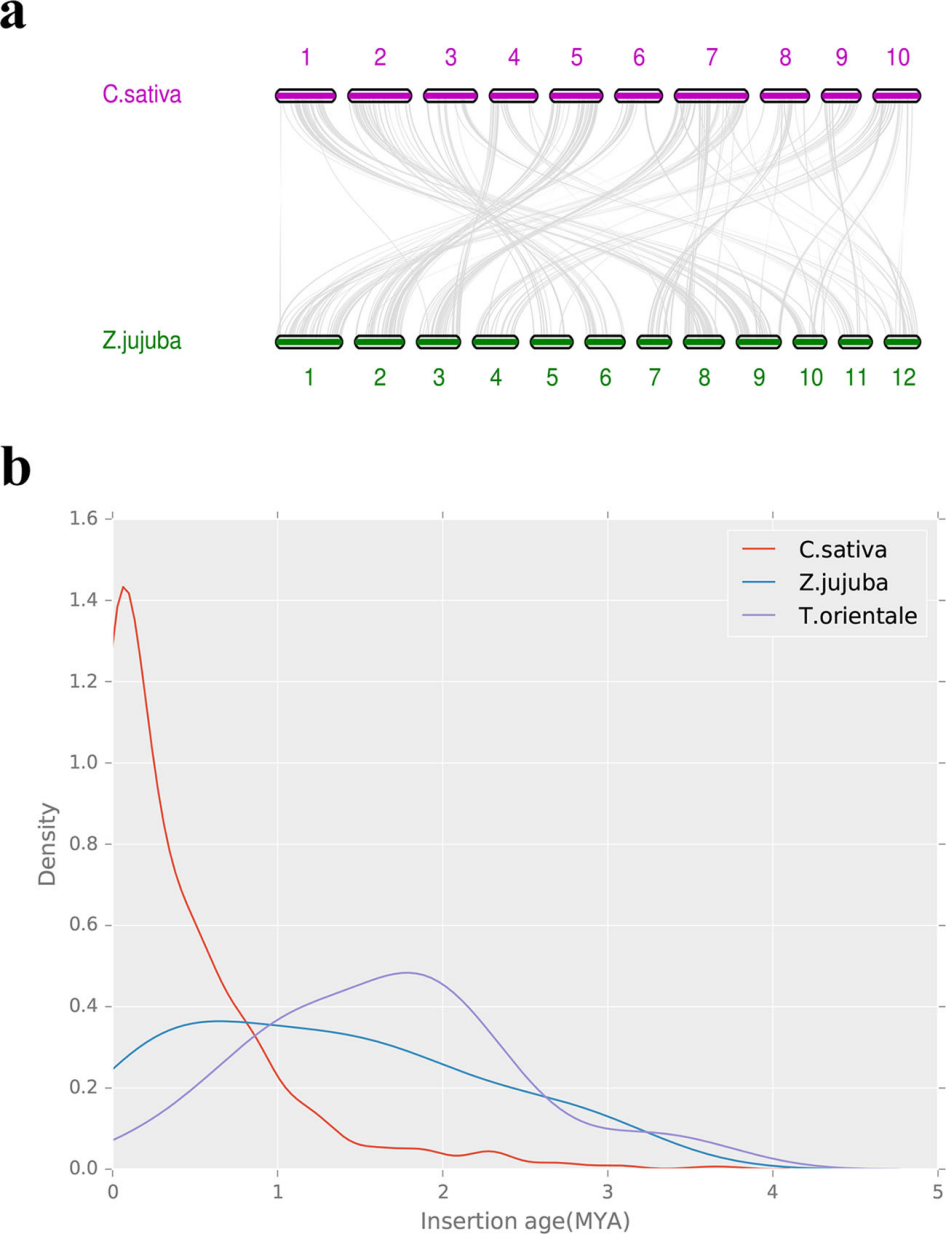
Synteny analysis and LTR analysis. **a** *C. sativa* and *Z. jujuba* synteny showing the span of their shared regions. **b** Long terminal repeat (LTR) occurrence time in different species

Genomic synteny block analysis can be used to determine the evolutionary source of chromosomes between species^38–40^. In this study, we used BLASTP (v2.2.31+) to analyze the aligned protein sequences of *C. sativa* and *Z. jujuba* (Rhamnaceae) and then used MCScan (v0.8) to evaluate those results by using genome synteny blocks. Our results showed that *C. sativa* and *Z. jujuba* present a strong genomic synteny relationship (Fig. 6a).

LTR recognition and the identification of occurrence history are important in plant genome research. In a plant genome, LTR retrotransposons are the type of transposon constituting the majority of plant TEs. The identification of the historical occurrence of such elements is important for plant genome research, thus placing much emphasis on characterizing LTR structure and identifying LTR sequences. At the same time, the study of LTR evolution elucidates the evolution of plant genome structure and function. LTRs are involved in shaping genomic structure and size, thus affecting the regulation and variation of genes and the origin of new genes. Since the discovery of LTR retrotransposons, research has shown that differences in LTR occurrence can be identified by comparing the time difference between sequenced, target species and related species^41^. In this study, LTRharvest (v1.5.10)^42^ and LTR_Finder (v1.0.5)^43^ were used to identify *de novo* LTR regions of *C. sativa*, *T. orientale*, and *Z. jujuba*. LTR_retriever (https://github.com/oushujun/LTR_retriever)^44^ was used to integrate the results from LTRharvest and LTR_Finder to obtain a high-quality LTR-RT library and to perform genome-wide LTR-RT annotation. We then generated LTR annotation regions and sequence information for the three species (Supplementary Information; Tables S11–S13). Through this LTR recognition, we found that the LTR retrotransposon content of 37.1% constituted most of the LTR TEs. Our estimation of LTR occurrence times revealed that *C. sativa*’s recent LTR burst differed from those of both *T. orientale* and *Z. jujuba* (Fig. 6b).

## Discussion

*C. sativa* is an invaluable plant species given its economic and ecological significance. Here, we generated an updated draft genome for wild-type varieties of *C. sativa* in China using PacBio single-molecule sequencing and Hi-C technology. Our assembled genome is approximately 808 Mb, with scaffold and contig N50 sizes of 83.00 Mb and 513.57 kb, respectively. Our new genome version is more contiguous than the previously assembled genome according to its a contig N50 of 12 kb. Therefore, the genome assembled in this study is superior to the previously assembled genome version.

In this study, we found a contig N50 of 632 kb for the PacBio read assembly. The genome continued to be assembled with the help of the Hi-C physical map, and the contig N50 became 513 kb. Here, we assumed that Hi-C technology concatenated the contigs into superlong scaffolds at the chromosome level. If the contigs were initially assembled correctly, the contig N50 would not change. However, due to the high heterozygosity of the wild-type varieties of *C. sativa*, chimeras were prone to occur during the assembly process, so the result of the assembly would be cut. Once the contig was cut, the contig N50 would decrease slightly, and we therefore considered the slight decrease in the contig N50 to be normal. Scaffolding with Hi-C further facilitated the assignment of all scaffolds to chromosomal positions. In this study, the new genome assembly presented higher genome contiguity and sequence quality than the previous genome assembly.

Because of the high repetition rate and high heterozygosity in the cannabis genome, no high-quality cannabis genome has been generated previously. There are unknown regions in the cannabis genome assembled using SOAPdenovo software by Van Bakel et al. in 2011^9^. The genome was not assembled to the chromosome level, and the number and length of the scaffolds in that study are much lower than the values expected for plant genes. Therefore, the cannabis genome assembled in 2011 shows poor quality and does not contain annotation information, which greatly limits its applicability to research on cannabis. In this study, we reassembled a wild-type cannabis genome by using third-generation sequencing data and thus obtained a high-quality cannabis genome.

Single-molecule real-time sequencing has the characteristics of a high throughput and long read length, which can reduce the number of contigs after sequencing and can effectively increase the number and integrity of genomes during the process of genome splicing. We combined TGS and NGS sequencing methods with Hi-C assembly technology to construct a high-density wild-type cannabis genome sequence map.

After obtaining the high-quality cannabis genome, we annotated its genes and thus considerably improved upon the 2011^9^ version of the genome. Following the completion of the assembly of its repeat sequences and statistical analysis, we found that cannabis has abundant repeat regions, which may be the cause of the poor quality of the cannabis genome assembled by Van Bakel et al. in 2011^9^. This high-quality reference genome will undoubtedly benefit researchers in the exploration and manipulation of the agronomic characteristics of *C. sativa*.

To understand the evolutionary status of cannabis, we analyzed its evolution and divergence times. Through these analysis, we found that the evolutionary status of cannabis and *T. orientale* is close at the molecular level, and their kinship is thus very close. The quality of the *T. orientale* genome is still relatively poor, and the high-quality cannabis genome that we obtained in this study could therefore provide useful information for the future study of the *T. orientale* genome and its evolution. By analyzing whole-genome duplication events in cannabis, we found three recent WGD events and one large-scale duplication event in cannabis and that cannabis shares two WGD incidents with *T. orientale*. Our data further elucidate the evolutionary status of cannabis.

## Materials and methods

### DNA extraction and sequencing

All samples were collected from the Kyirong Gully (28°28′–28°66′ N, 85°13′–85°28′ E) in Tibet, China. The Kyirong Gully is a plateau gorge with an altitude of 1,700–6,000 m located on the south slope of the Himalayas and is very isolated from the outside world. Its special geography and environment make the gorge a typical vertical ecological system of Himalayan areas, and it is considered “the richest species gene bank in a plateau area”. We extracted high-quality genomic DNA from the leaves of female *C. sativa* using a Plant DNA kit (TIANGEN Biotech, Beijing, China). A 10–20 kb SMRTbell DNA library (Pacific Biosciences of California, CA, USA)^45^ was prepared using BluePippin for DNA size selection (Sage Science, MA, USA) and then sequenced on the PacBio Sequel platform (Pacific Biosciences of California, CA, USA) with P6/C4 chemistry. Finally, 124 Gb of subreads were obtained, with 153× coverage of the *C. sativa* genome.

A paired-end library with an insert size of 350 bp was constructed and sequenced using the Illumina HiSeq X Ten platform.

Total RNA was extracted from the roots, stems, leaves, and seeds using the QIAGEN Total RNA Extraction Kit (QIAGEN, Hilden, Germany), and RNA-seq libraries were then constructed using the TruSeq RNA Library Kit (Illumina, CA, USA). These data were used for gene structure prediction.

### Genome assembly

Since PacBio single-molecule sequencing usually shows an unacceptably high error rate, we used Canu^46^ (v1.5) to perform self-correction before assembly. A variety of long-read assemblers, such as SMARTdenovo (v1.5)^47^ and Wtdgb2 (v2.1), were employed for *C. sativa* contig construction using the corrected subreads. To further improve contig continuity, the QuickMerge meta-assembler (https://github.com/mahulchak/quickmerge)^48^ was employed with the contigs from Wtdbg2 as the query input and those from SMARTdenovo (v1.5) as the ref input. The two sets of contigs were aligned using NUCmer from MUMmer (v4.0.0) with the parameters “-l 100” and the delta-filter parameters “-i 95 -r”. Then, QuickMerge (https://github.com/mahulchak/quickmerge) was used with the parameters ‘-hco 5.0 -c 1.5 -lm 5000’. The errors in the primary assembly were corrected by using PacBio subreads with blasr (v5.1) and Arrow (v2.2.1), and the Illumina paired-end reads were then mapped to the contigs using bwa-mem to polish the contigs with Pilon (v1.22, Broad Institute, MA, USA)^13^.

### Evaluation of the assembled genome

Two methods were used to evaluate the quality of the final assembly: (1) We used the Benchmarking Universal Single-Copy Orthologs (BUSCO v3.0)^18^ approach to evaluate the accuracy and completeness of the genome assembly, which provides quantitative measures for the assessment of genome assembly based on evolutionarily informed expectations of gene content from near-universal single-copy orthologs; and (2) Illumina paired-end reads were mapped to the final assembly to evaluate its completeness using bwa-mem with the default parameters.

### Chromosome assembly using Hi-C

In this study, we used *C. sativa* root samples for Hi-C experiments and data analysis, and leaf samples from the same plant were used for to generate a Hi-C library. The Hi-C library was prepared using a NEBNext Ultra II DNA Library Prep Kit, and the library was sequenced on the Illumina HiSeq X Ten platform.

The clean reads were mapped to the *C. sativa* genome using Bowtie2 (v2.2.3) with the default parameters. The two ends of paired reads were separately mapped to the genome. After filtering out dangling ends, self-annealing sequences, and dumped pairs, the valid paired-end reads of unique, mapped paired-end reads were collected using HiC-Pro (v2.10)^49^. Finally, we applied the agglomerative hierarchical clustering method in Lachesis (https://github.com/shendurelab/LACHESIS)^17^. Valid interaction pairs were used to build interaction matrices and scale up the primary contigs to chromosome-scale scaffolds with LACHESIS^17^. The general procedure of LACHESIS is to first cluster the contigs into chromosomal groups with the agglomerative hierarchical clustering algorithm and then order and orient the contigs of each chromosomal group into pseudochromosomes. We set CLUSTER_N=10 for LACHESIS, and then performed full-range scanning of the five key parameters, including CLUSTER_MIN_RE_SITES [15, 2000], CLUSTER_MAX_LINK_DENSITY [1, 10], CLUSTER_NONINFORMATIVE_RATIO [1v, 10], ORDER_MIN_N_RES_IN_TRUNK [15, 2000], and ORDER_MIN_N_RES_IN_SHREDS [15, 2000]. After approximately 1,000 trials, the best candidate was selected if it included >95% of the ordered contig length and rearranged manually. Finally, 2,506 contigs (representing 99.8% of the total length) were anchored to 10 pseudochromosomes of *C. sativa*.

### Assessment of genomic integrity

We used BUSCO (v3.0)^18^ to evaluate the accuracy and completeness of our genome assembly, gene set, and transcripts. Based on the OrthoDB (http://cegg.unige.ch/orthodb) database, BUSCO builds several large, single-copy gene sets covering branches of the evolutionary tree. When comparing the gene set to the genome, we found that the proportion of complete BUSCOs was 92.6%, thus indicating very good genome assembly integrity.

### Repeat element identification

Repeat sequences, including tandem repeats and interspersed repeats, are important components of the genome. Two strategies, homology alignment and *de novo* searches, were combined to identify repeats. First, we identified homologous repeat sequences based on RepBase (https://www.girinst.org/server/RepBase/index.php)^50^. In addition, we used the *de novo* prediction method of RepeatModeler (http://www.repeatmasker.org/RepeatModeler/) with the default parameters. Tandem Repeat Finder (https://tandem.bu.edu/trf/trf.html) was used to find tandem repeats in the genome.

LTR-RTs are important for plant genome evolution, so we used LTRharvest^42^ and LTR_Finder^43^ to identify the *de novo* LTRs of *C. sativa*, *T. orientale* and *Z. jujuba*. LTR_retriever^44^ was used to integrate the results from LTRharvest and LTR_Finder to obtain a high-quality LTR-RT library and perform genome-wide LTR-RT annotation.

### Noncoding RNA prediction

tRNA genes were detected with tRNAscan-SE (http://lowelab.ucsc.edu/tRNAscan-SE/), and other noncoding RNAs, such as rRNAs, tRNAs, snRNAs, and miRNAs, were predicted by comparison with the known noncoding RNA library of the Rfam database (http://rfam.xfam.org/).

### Gene prediction

Gene models were predicted by integrating three approaches: homology-based, transcriptome-based, and ab initio prediction. In homology-based prediction, the protein sequences of *Arabidopsis thaliana*, *Oryza sativa*, and *Zea mays* were downloaded and aligned to the genome using BLAST (*E*-value: 1e − 5), and gene models were defined using GeMoMa^51^. CPC^19^ was used to examine gene coding potential. A gene model was retained as the final set if it presented evidence of transcription and coding potential.

### Functional annotation

We predicted the function of the protein-coding gene set by searching against public databases including SwissProt (http://www.ebi.ac.uk/interpro/search/sequence-search), NT (https://www.ncbi.nlm.nih.gov/nucleotide/), NR (https://www.ncbi.nlm.nih.gov/protein/), PFAM (http://xfam.org/)^20^, eggNOG (http://eggnogdb.embl.de)^21^, GO (http://geneontology.org/page/go-database)^22^, and KEGG (http://www.genome.jp/kegg/)^23^.

### Gene families and phylogenetic analysis

The genomes of *C. sativa* and seven other plants, including *Z. jujube, V. vinifera, F. vesca, P. somniferum, T. orientate, M. notabilis*, and *M. domestica*, were collected for evolutionary analysis. An all-vs.-all BLASTP (v2.2.28) (*E*-value: 1e − 5) search was carried out, and then OrthoMCL^26^ was used to identify paralogous and orthologous genes.

MUSCLE^27^ was used to perform multiple sequence alignments for all single-copy orthologous genes. After we constructed the integrated supergene sequence, which was based on the four-fold degenerate sites (4DTv sites) of single-copy orthologous genes, PhyML^28^ was used to construct the phylogenetic tree (ML-Tree) with bootstrap values. The divergence time between the eight species were estimated using MCMCTREE^29^ of the PAML package. The calibration points were selected from the TimeTree database (http://www.timetree.org/)^30–33^ as the normal priors to restrain the age of the nodes.

### Expansion/contraction analysis of gene families

After gene families cluster analysis, the gene families sizes from OrthoMCL and the phylogenetic trees, including branch lengths, were used as inputs for CAFE^34^. The λ value was estimated based on a stochastic birth and death process model. Gene families were considered significantly expanded or contracted when they presented *p* values smaller than 0.05.

### Synteny and whole-genome duplication

Whole-genome duplication events are widespread in plants and are important for dynamic genome evolution. MCscan^35,36^ was used to identify synteny blocks, defined as regions with more than five collinear genes between paired genomes. MUSCLE^27^ was used to perform multiple sequence alignment for the sequences of the synteny blocks. The 4DTv value was calculated as the number of transversions at all four-fold degenerate synonymous sites.

## Supplementary information

Table S1: Summary of the transcriptomes

Table S2: Results of base content in the genome

Table S3: Summary of BUSCO evaluation results

Table S4: Results of genomic consistency assessment

Table S5: Statistical results of genomic repeat sequencing

Table S6: Statistical results of genomic repeat sequencing classification

Table S7: Statistics of non-coding RNAs

Table S8: Results of BUSCO evaluation of genes

Table S9: Quantitative statistics of gene clustering to family

Table S10: time_correction

Table S11: Cannabis_sativa_nmtf_pass

Table S12: Trema_orientale_nmtf_pass

Table S13: Ziziphus_jujuba_nmtf_pass

Figure S1

Figure S2

Figure S3

Figure S4

Figure S5

Figure S6

## Data Availability

The *C. sativa* genome project data were deposited at NCBI under BioProject number PRJNA562042 and BioSamples SAMN12606152 and SAMN12855826. The DNAseq clean data, RNA-seq clean data, and Hi-C clean data of the wild Chinese *C. sativa* genome have been deposited in the Sequence Read Archive (SRA) database under accession numbers SRR10019825 and SRR10193436–SRR10193442. The *de novo* clean data of the wild *C. sativa* genome have been deposited in the Sequence Read Archive (SRA) database under accession numbers SRR10189115–SRR10189120. Detailed information on the genome can be found on the NCBI platform (https://dataview.ncbi.nlm.nih.gov/object/PRJNA562042?reviewer=h2q2tfglbtejieim9o430aan6n). The data from this whole-genome project have been deposited at DDBJ/ENA/GenBank under accession number WRXK00000000. The version described in this paper is version WRXK01000000 under BioProject number PRJNA562042 and BioSample SAMN12606152. All supplementary figures and tables are provided in the Supplementary Materials.

## Code availability

**(1) FALCON:** version 3.1, length_cutoff=5000, length_cutof_pr=5000, max_dif=120, max_cov=130; **(2) Canu:** version 1.5, corOutCoverage=80; **(3) SmartDenovo:** version 1.5, default parameters; **(4) WTDBG:** version 2.2, p 21, S 4, s 0.05, L 5000; **(5) Quickmerge:** version 0.2, l 100, -ml 5000; **(6) Blasr:** version 5.1, -bam, -bestn 5, -minMatch 18, -minSubreadLength 1000, -minAlnLength 500; **(7) Arrow:** version 2.2.1, default parameters; **(8) BWA:** version 0.7.9a, mem -M -k 30; **(9) Pilon:** version 1.22, default parameters; **(10) BUSCO:** version 3.0.1, default parameters, Embryophyta Version odb10; **(11) RepeatMasker:** version 1.323, e ncbi; **(12) RepeatProteinMask:** version 1.36, default parameters; **(13) RepeatModeler:** version open-1.0.8, -engine ncbi; http://www.repeatmasker.org/RepeatModeler/; **(14) GeMoMa:** version 1.0, default parameters; **(15) CPC:** version 0.9-r2, default parameters; **(16) HMMER:** version 3.1b1, default parameters; **(17) TRINOTATE:** version 3.0.2, default parameters; **(18) Blast:** version 2.2.28, evalue=1e^−5^; **(19) tRNAscan-SE:** version 1.3.1, -X 20, z 8; http://lowelab.ucsc.edu/tRNAscan-SE/; **(20) InterProScan:** version 4.8, default parameters; **(21) NR database:** version 08-10-2015; ftp://ftp.ncbi.nlm.nih.gov/blast/db/FASTA/nr.gz**; (22) KEGG database:** version 08-31-2015; http://www.genome.jp/kegg/**; (23) SwissProt database:** version 05-24-2016; https://web.expasy.org/docs/swiss-prot_guideline.html; **(24) Rfamdatabase:** version 14.1; **(25) Pfam database:** version 03-30-2016; http://xfam.org/; **(26) OrthoMCL:** version 1.4,default parameters; http://OrthoMCL.org/OrthoMCL/; **(27) MUSCLE:** version v3.8.31, maxiters 16; http://www.drive5.com/muscle/; **(28) PhyML:** version 3.0,default parameters; **(29) MCMCtree:** version 4.4, burn-in=20000, sample-frequency=2; **(30) PAML:** version 4.9, default parameters; http://abacus.gene.ucl.ac.uk/software/paml.html; **(31) CAFE:** version 4.1, default parameters; http://sourceforge.net/projects/cafehahnlab/; **(32) MCScan:** version 0.8, -g 3, e 1e-05, -u 10000; http://chibba.agtec.uga.edu/duplication/mcscan/; **(33) LTR_FINDER:** version 1.0.5, default parameters; **(34) LTR_harverst:** version 1.5.10, default parameters; **(35) LTR_retriever:** version 1.0, default parameters.

## References

[1] Schultes RE, Klein WM, Plowman T, Lockwood TE (1974). Cannabis: an example of taxonomic neglect. Bot. Mus. Leafl., Harv. Univ..

[2] Li H-L (1973). An archaeological and historical account of cannabis in China. Econ. Bot..

[3] Leung L (2011). Cannabis and its derivatives: review of medical use. J. Am. Board Fam. Med..

[4] Ruiz L, Miguel A, Díaz-Laviada I (1999). Δ9‐Tetrahydrocannabinol induces apoptosis in human prostate PC‐3 cells via a receptor‐independent mechanism. FEBS Lett..

[5] Esposito G, De Filippis D, Carnuccio R, Izzo AA, Iuvone T (2006). The marijuana component cannabidiol inhibits β-amyloid-induced tau protein hyperphosphorylation through Wnt/β-catenin pathway rescue in PC12 cells. J. Mol. Med..

[6] Martín-Moreno AM (2011). Cannabidiol and other cannabinoids reduce microglial activation in vitro and in vivo: relevance to Alzheimers′ disease. Mol. Pharmacol..

[7] Steffens S (2005). Low dose oral cannabinoid therapy reduces progression of atherosclerosis in mice. Nature.

[8] Taura F, Sirikantaramas S, Shoyama Y, Shoyama Y, Morimoto S (2007). Phytocannabinoids in *Cannabis sativa*: recent studies on biosynthetic enzymes. Chem. Biodivers..

[9] Van Bakel H (2011). The draft genome and transcriptome of *Cannabis sativa*. Genome Biol..

[10] Ming R, Bendahmane A, Renner SS (2011). Sex chromosomes in land plants. Annu. Rev. Plant Biol..

[11] Sakamoto K, Akiyama Y, Fukui K, Kamada H, Satoh S (1998). Characterization; genome sizes and morphology of sex chromosomes in hemp (*Cannabis sativa* L.). Cytologia.

[12] Li YH (2014). De novo assembly of soybean wild relatives for pan-genome analysis of diversity and agronomic traits. Nat. Biotechnol..

[13] Walker BJ (2014). Pilon: an integrated tool for comprehensive microbial variant detection and genome assembly improvement. PLoS One.

[14] Lieberman-Aiden E (2009). Comprehensive mapping of long-range interactions reveals folding principles of the human genome. Science.

[15] Rao SS (2014). A 3D map of the human genome at kilobase resolution reveals principles of chromatin looping. Cell.

[16] Belaghzal H, Dekker J, Gibcus JH (2017). Hi-C 2.0: An optimized Hi-C procedure for high-resolution genome-wide mapping of chromosome conformation. Methods.

[17] Burton JN (2013). Chromosome-scale scaffolding of de novo genome assemblies based on chromatin interactions. Nat. Biotechnol..

[18] Simao FA, Waterhouse RM, Ioannidis P, Kriventseva EV, Zdobnov EM (2015). BUSCO: assessing genome assembly and annotation completeness with single-copy orthologs. Bioinformatics.

[19] Kong L (2007). CPC: assess the protein-coding potential of transcripts using sequence features and support vector machine. Nucleic Acids Res..

[20] Finn RD (2014). Pfam: the protein families database. Nucleic Acids Res..

[21] Powell S (2012). eggNOG v3.0: orthologous groups covering 1133 organisms at 41 different taxonomic ranges. Nucleic Acids Res..

[22] Ashburner M (2000). Gene ontology: tool for the unification of biology. The Gene Ontology Consortium. Nat. Genet..

[23] Kanehisa M (2013). Molecular network analysis of diseases and drugs in KEGG. Methods Mol. Biol..

[24] Griffiths-Jones S (2005). Rfam: annotating non-coding RNAs in complete genomes. Nucleic Acids Res..

[25] Lowe TM, Eddy SR (1997). tRNAscan-SE: a program for improved detection of transfer RNA genes in genomic sequence. Nucleic Acids Res..

[26] Li L, Stoeckert CJ, Roos DS (2003). OrthoMCL: identification of ortholog groups for eukaryotic genomes. Genome Res..

[27] Edgar RC (2004). MUSCLE: multiple sequence alignment with high accuracy and high throughput. Nucleic Acids Res..

[28] Guindon S (2010). New algorithms and methods to estimate maximum-likelihood phylogenies: assessing the performance of PhyML 3.0. Syst. Biol..

[29] Yang Z (2007). PAML 4: phylogenetic analysis by maximum likelihood. Mol. Biol. Evol..

[30] Kumar S, Stecher G, Suleski M, Hedges SB (2017). TimeTree: a resource for timelines, timetrees, and divergence times. Mol. Biol. Evol..

[31] Zeng Q (2015). Definition of eight mulberry species in the genus morus by internal transcribed spacer-based phylogeny. PLoS One.

[32] Foster CSP (2017). Evaluating the impact of genomic data and priors on Bayesian estimates of the angiosperm evolutionary timescale. Syst. Biol..

[33] Massoni J, Couvreur TL, Sauquet H (2015). Five major shifts of diversification through the long evolutionary history of Magnoliidae (angiosperms). BMC Evolut. Biol..

[34] De Bie T, Cristianini N, Demuth JP, Hahn MW (2006). CAFE: a computational tool for the study of gene family evolution. Bioinformatics.

[35] Huang S (2009). The genome of the cucumber, *Cucumis sativus* L. Nat. Genet..

[36] Schmutz J (2010). Genome sequence of the palaeopolyploid soybean. Nature.

[37] Jaillon O (2007). The grapevine genome sequence suggests ancestral hexaploidization in major angiosperm phyla. Nature.

[38] Tang H (2008). Synteny and collinearity in plant genomes. Science.

[39] Soderlund C, Bomhoff M, Nelson WM (2011). SyMAP v3.4: a turnkey synteny system with application to plant genomes. Nucleic Acids Res..

[40] Dujon B (2004). Genome evolution in yeasts. Nature.

[41] Shi J (2019). Chromosome conformation capture resolved near complete genome assembly of broomcorn millet. Nature.

[42] Ellinghaus D, Kurtz S, Willhoeft U (2008). LTRharvest, an efficient and flexible software for de novo detection of LTR retrotransposons. BMC Bioinform..

[43] Xu Z, Wang H (2007). LTR_FINDER: an efficient tool for the prediction of full-length LTR retrotransposons. Nucleic Acids Res..

[44] Ou S, Jiang N (2018). LTR_retriever: a highly accurate and sensitive program for identification of long terminal repeat retrotransposons. Plant Physiol..

[45] English AC (2012). Mind the gap: upgrading genomes with Pacific Biosciences RS long-read sequencing technology. PLoS One.

[46] Koren S (2017). Canu: scalable and accurate long-read assembly via adaptive k-mer weighting and repeat separation. Genome Res..

[47] Istace B (2017). De novo assembly and population genomic survey of natural yeast isolates with the Oxford Nanopore MinION sequencer. GigaScience.

[48] Chakraborty M, Baldwin-Brown JG, Long AD, Emerson JJ (2016). Contiguous and accurate de novo assembly of metazoan genomes with modest long read coverage. Nucleic Acids Res..

[49] Servant N (2012). HiTC: exploration of high-throughput ‘C’ experiments. Bioinformatics.

[50] Jurka J (2005). Repbase Update, a database of eukaryotic repetitive elements. Cytogenet. Genome Res..

[51] Keilwagen J (2016). Using intron position conservation for homology-based gene prediction. Nucleic Acids Res..

