# Supplementary material for: A high-quality reference genome of wild Cannabis sativa
Source: Hortic Res. 2020 May 2;7:73. doi: 10.1038/s41438-020-0295-3 (PMC7195422; doi:10.1038/s41438-020-0295-3)
Supplement: Supplementary file 1 — Table S1: Summary of the transcriptomes [file 41438_2020_295_MOESM1_ESM.docx]

Table1: Summary of the transcriptomes

| Sample | Stem1-2-1 | Leaf-1 | Seed1-1 | Root1-2 | Root1-3 |
| --- | --- | --- | --- | --- | --- |
| Clean Bases Number | 9,286,890,900 | 9,656,339,700 | 9,766,120,200 | 6,518,546,400 | 6,779,039,400 |
