# Supplementary material for: A high-quality reference genome of wild Cannabis sativa
Source: Hortic Res. 2020 May 2;7:73. doi: 10.1038/s41438-020-0295-3 (PMC7195422; doi:10.1038/s41438-020-0295-3)
Supplement: Supplementary file 2 — Table S2: Results of base content in the genome [file 41438_2020_295_MOESM2_ESM.docx]

Table 2: Results of base content in the genome

| Iterms | Number | Percent(%) |
| --- | --- | --- |
| A | 268,963,633 | 33.11% |
| T | 268,803,913 | 33.09% |
| C | 137,221,748 | 16.89% |
| G | 137,306,526 | 16.9% |
| N | 0 | 0.0% |
| GC | 274,528,274 | 33.8% |
| Total Genome base | 812,295,820 | - |
