# Supplementary material for: A high-quality reference genome of wild Cannabis sativa
Source: Hortic Res. 2020 May 2;7:73. doi: 10.1038/s41438-020-0295-3 (PMC7195422; doi:10.1038/s41438-020-0295-3)
Supplement: Supplementary file 3 — Table S3: Summary of BUSCO evaluation results [file 41438_2020_295_MOESM3_ESM.docx]

Table 3: Summary of BUSCO evaluation results

| Iterms | Number | Percent (%) |
| --- | --- | --- |
| Complete BUSCOs (C) | 1274 | 92.6 |
| Complete and single-cope BUSCO (S) | 1062 | 77.2 |
| Complete and duplicated BUSCO (D) | 212 | 15.4 |
| Fragmented BUSCO (F) | 12 | 0.9 |
| Missing BUSCO (M) | 89 | 6.5 |
| Total BUSCO groups searched | 1375 | 100 |
