# Supplementary material for: A high-quality reference genome of wild Cannabis sativa
Source: Hortic Res. 2020 May 2;7:73. doi: 10.1038/s41438-020-0295-3 (PMC7195422; doi:10.1038/s41438-020-0295-3)
Supplement: Supplementary file 4 — Table S4: Results of genomic consistency assessment [file 41438_2020_295_MOESM4_ESM.docx]

Table 4: Results of genomic consistency assessment

| Sample | JL |
| --- | --- |
| Clean Reads | 639,815,862 |
| Clean Bases | 95,972,379,300 |
| Mapped Reads | 628,068,306 |
| Mapped Reads Rate (%) | 98.16 |
| Mapped Bases | 92,871,813,877 |
| Mapped Bases Rate (%) | 96.77 |
| Mean Depth | 65.35 |
| Coverage Rate (%) | 88.76 |
