# Supplementary material for: A high-quality reference genome of wild Cannabis sativa
Source: Hortic Res. 2020 May 2;7:73. doi: 10.1038/s41438-020-0295-3 (PMC7195422; doi:10.1038/s41438-020-0295-3)
Supplement: Supplementary file 5 — Table S5: Statistical results of genomic repeat sequencing [file 41438_2020_295_MOESM5_ESM.docx]

Table 5: Statistical results of genomic repeat sequencing

| Type | Repeat length (bp) | % of genome |
| --- | --- | --- |
| RepeatMasker | 118,700,582 | 14.61 |
| ProteinMask | 161,847,743 | 19.92 |
| Denovo | 584,319,477 | 71.93 |
| Trf | 22,382,718 | 2.76 |
| Total | 612,733,451 | 74.75 |
