# Supplementary material for: A high-quality reference genome of wild Cannabis sativa
Source: Hortic Res. 2020 May 2;7:73. doi: 10.1038/s41438-020-0295-3 (PMC7195422; doi:10.1038/s41438-020-0295-3)
Supplement: Supplementary file 6 — Table S6: Statistical results of genomic repeat sequencing classification [file 41438_2020_295_MOESM6_ESM.docx]

Table 6: Statistical results of genomic repeat sequencing classification

| Class |  | RepeatMask er TEs |  | RepeatProte InMask TEs |  | RepeatMode  Ler TEs |  | Combined TEs |
| --- | --- | --- | --- | --- | --- | --- | --- | --- |
| Type | Length(bp) | % In genome | Length(bp) | % In genome | Length(bp) | % In genome | Length(bp) | % In genome |
| DNA | 5,500,071 | 0.68 | 2,276,264 | 0.28 | 24,305,045 | 2.99 | 26,532,025 | 3.27 |
| LINE | 5,133,696 | 0.63 | 13,533,723 | 1.67 | 30,869,107 | 3.80 | 33,429,432 | 4.12 |
| SINE | 2,689 | 0.00 | 0 | 0.00 | 0 | 0.00 | 2,689 | 0.00 |
| LTR | 106,478,213 | 13.11 | 118,210,705 | 14.55 | 391,892,927 | 48.25 | 406,162,228 | 50.00 |
| Unknown | 55,349 | 0.01 | 0 | 0.00 | 142,250,202 | 17.51 | 142,297,890 | 17.52 |
| Other | 2,004,030 | 0.25 | 27,971,757 | 3.44 | 7,813,489 | 0.96 | 35,263,677 | 4.34 |
| Total | 118,700,582 | 14.61 | 161,847,743 | 19.92 | 584,319,477 | 71.93 | 607,217,621 | 74.75 |
