# Supplementary material for: A high-quality reference genome of wild Cannabis sativa
Source: Hortic Res. 2020 May 2;7:73. doi: 10.1038/s41438-020-0295-3 (PMC7195422; doi:10.1038/s41438-020-0295-3)
Supplement: Supplementary file 7 — Table S7: Statistics of non-coding RNAs [file 41438_2020_295_MOESM7_ESM.docx]

Table 7: Statistics of non-coding RNAs

| Class | Type | Copy | Average lenth(bp) | Total length(bp) | %of genome |
| --- | --- | --- | --- | --- | --- |
| miRNA | miRNA | 281 | 139.72 | 39261 | 0.00483 |
| tRN | tRNA | 701 | 74.86 | 52376 | 0.00646 |
|  | 18S | 11 | 488.64 | 5375 | 0.00066 |
| rRNA | 28S | 17 | 139.71 | 2375 | 0.00029 |
|  | 5.8S | 5 | 139.00 | 695 | 0.00009 |
|  | 5S | 83 | 93.00 | 7719 | 0.00095 |
|  | CD-box | 2336 | 105.08 | 245472 | 0.03022 |
| snRNA | HACA-box | 83 | 123.90 | 10284 | 0.00127 |
|  | splicing | 131 | 138.53 | 18148 | 0.00223 |
