# Supplementary material for: A high-quality reference genome of wild Cannabis sativa
Source: Hortic Res. 2020 May 2;7:73. doi: 10.1038/s41438-020-0295-3 (PMC7195422; doi:10.1038/s41438-020-0295-3)
Supplement: Supplementary file 8 — Table S8: Results of BUSCO evaluation of genes [file 41438_2020_295_MOESM8_ESM.docx]

Table 8: Results of BUSCO evaluation of genes

| Iterms | Number | Percent (%) |
| --- | --- | --- |
| Complete BUSCOs (C) | 1282 | 93.2 |
| Complete and single-cope BUSCO (S) | 1049 | 76.3 |
| Complete and duplicated BUSCO (D) | 233 | 16.9 |
| Fragmented BUSCO (F) | 10 | 0.7 |
| Missing BUSCO (M) | 83 | 6.1 |
| Total BUSCO groups searched | 1375 | 100 |
