# Supplementary material for: A high-quality reference genome of wild Cannabis sativa
Source: Hortic Res. 2020 May 2;7:73. doi: 10.1038/s41438-020-0295-3 (PMC7195422; doi:10.1038/s41438-020-0295-3)
Supplement: Supplementary file 9 — Table S9: Quantitative statistics of gene clustering to family [file 41438_2020_295_MOESM9_ESM.docx]

Table 9: Quantitative statistics of gene clustering to family

| Species | Genes number | Genes in families | Unclustered genes | Family number | Unique families | Average genes per family |
| --- | --- | --- | --- | --- | --- | --- |
| C.sativa | 38828 | 36136 | 2692 | 14093 | 930 | 2.56 |
| M.notabilis | 26965 | 20385 | 6580 | 14207 | 657 | 1.43 |
| T.orientale | 32495 | 22477 | 10018 | 15054 | 744 | 1.49 |
| V.vinifera | 25001 | 22826 | 2175 | 13436 | 371 | 1.70 |
| F.vesca | 23791 | 21382 | 2409 | 13805 | 493 | 1.55 |
| M.domestica | 41955 | 32924 | 9031 | 14208 | 1473 | 2.32 |
| Z.jujuba | 28879 | 25812 | 3067 | 13767 | 593 | 1.87 |
| O.poppy | 62575 | 58720 | 3855 | 14371 | 2669 | 4.09 |
