# Supplementary figures and images for: A high-quality reference genome of wild Cannabis sativa
Source: Hortic Res. 2020 May 2;7:73. doi: 10.1038/s41438-020-0295-3 (PMC7195422; doi:10.1038/s41438-020-0295-3)

JL resolution=500000  
Genome-wide all-by-all Hi-C interaction

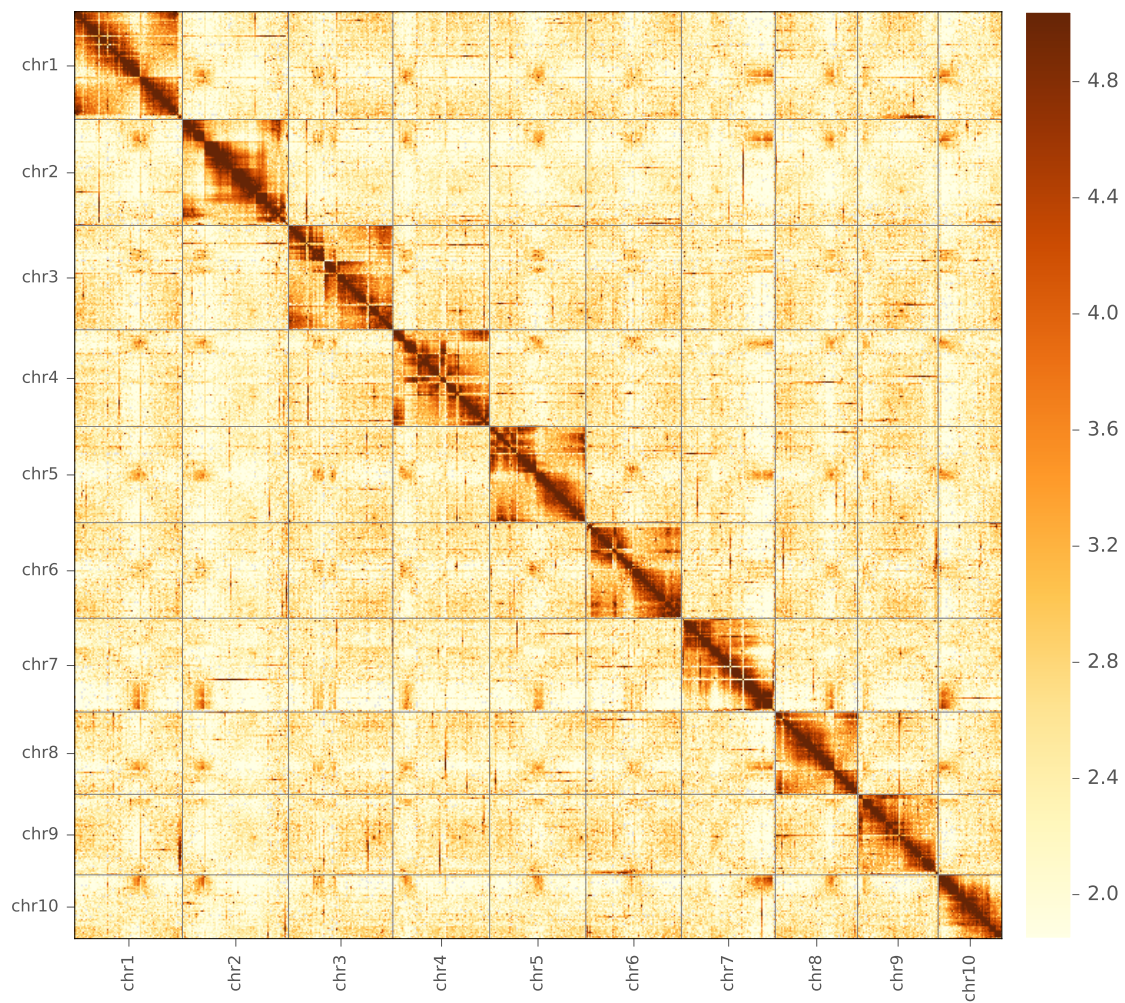

Supplement: Supplementary file 14 — Figure S1 [file 41438_2020_295_MOESM14_ESM.pdf]

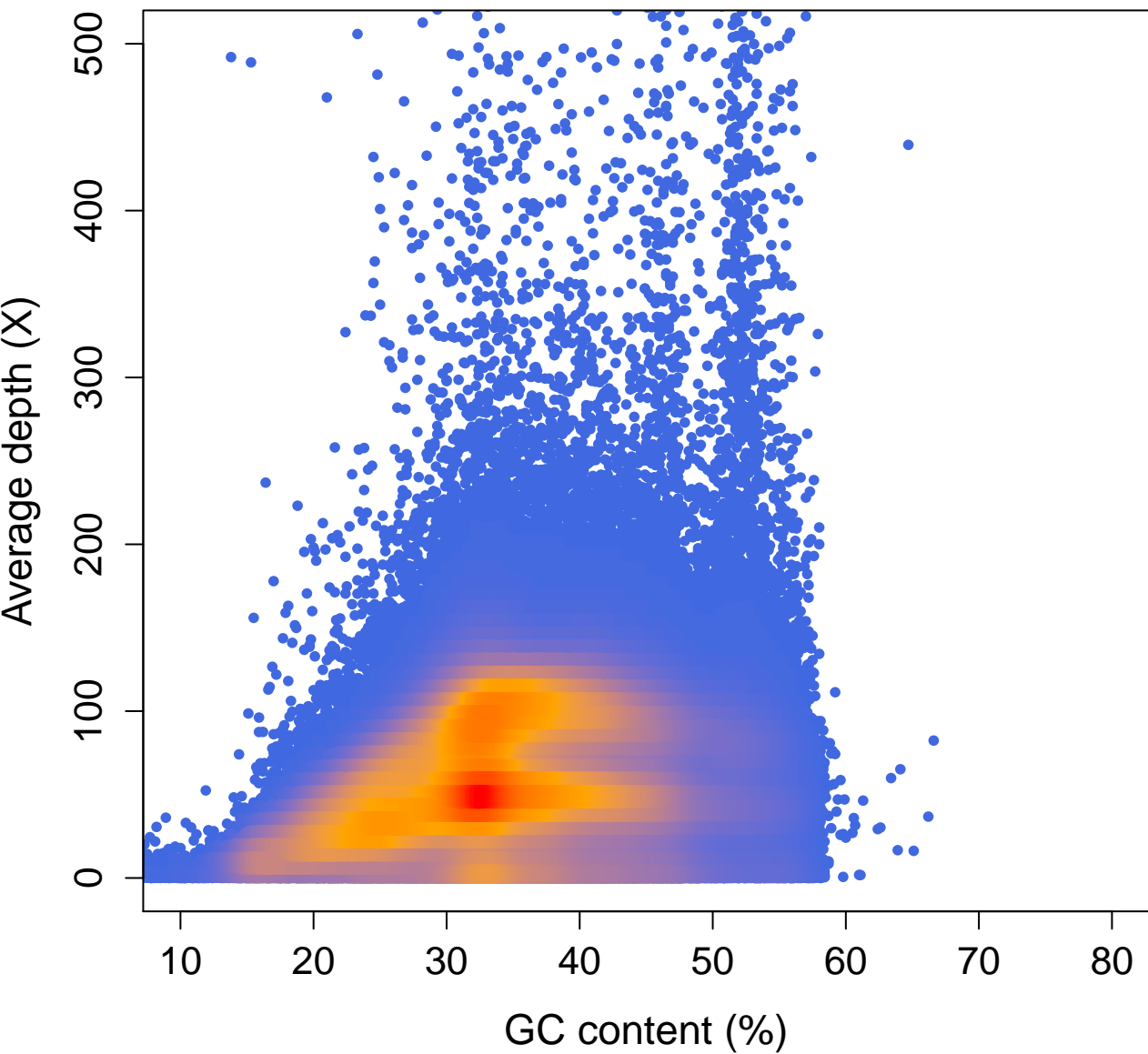

Supplement: Supplementary file 15 — Figure S2 [file 41438_2020_295_MOESM15_ESM.pdf]

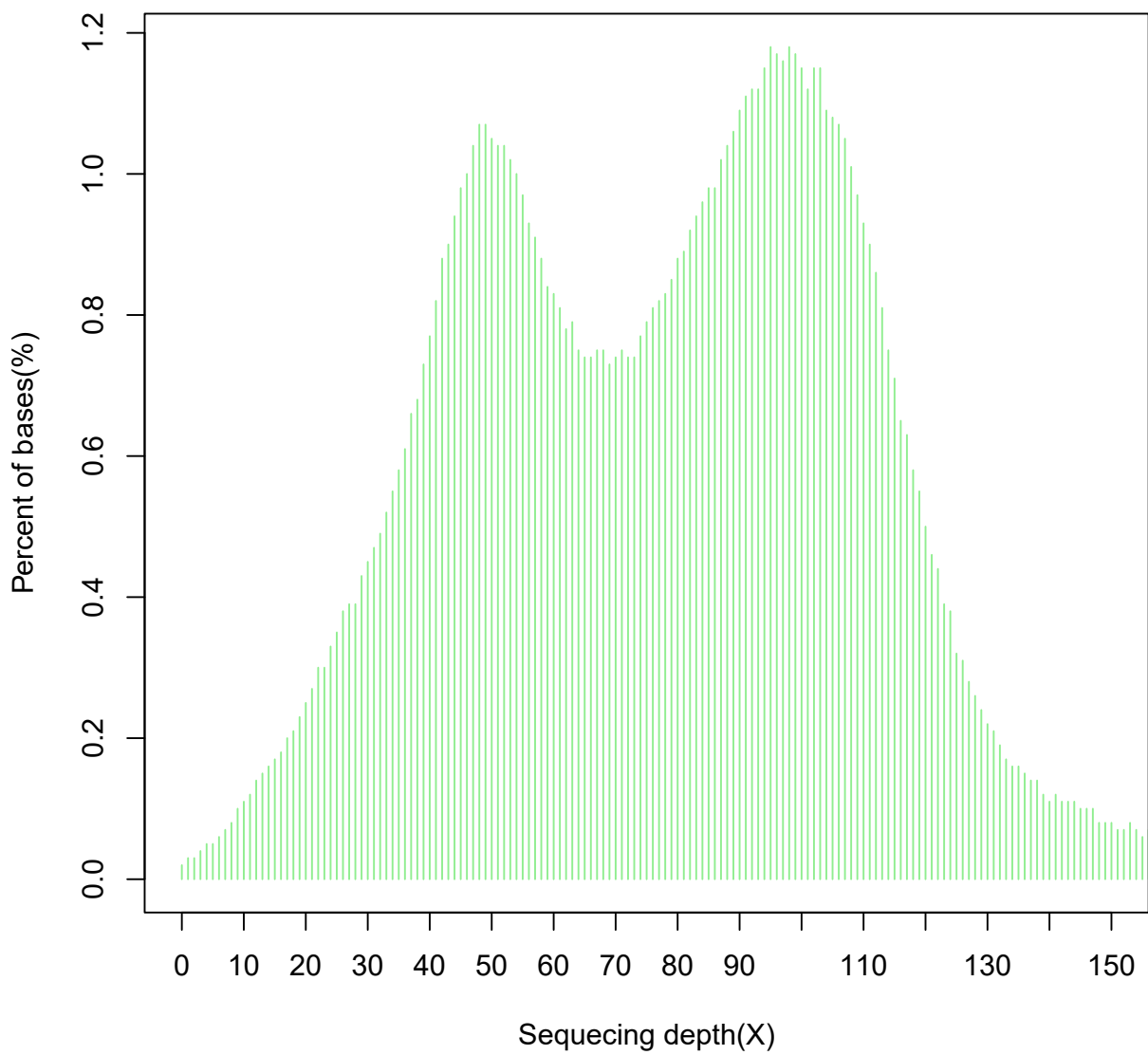

Supplement: Supplementary file 16 — Figure S3 [file 41438_2020_295_MOESM16_ESM.pdf]

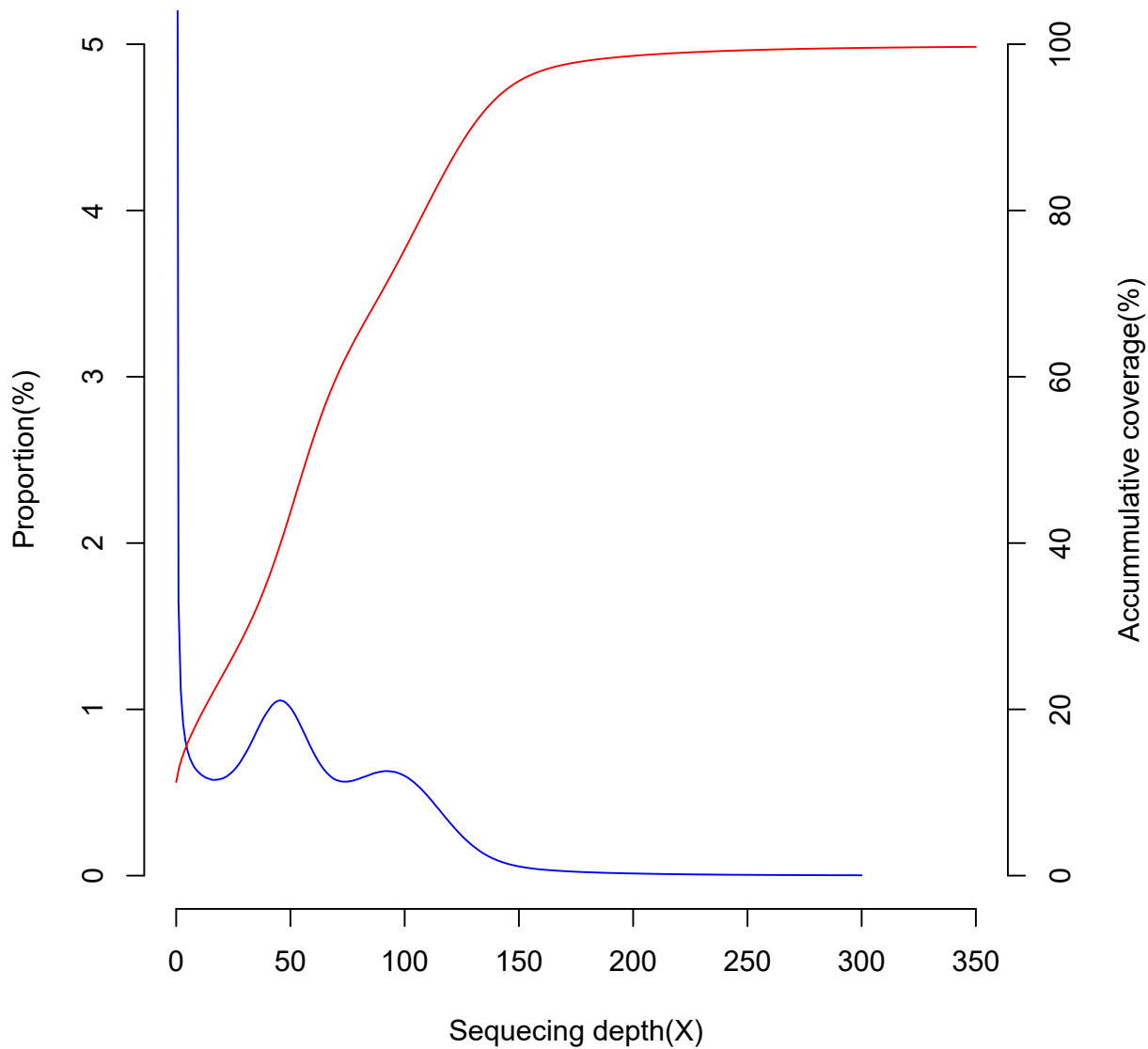

Supplement: Supplementary file 17 — Figure S4 [file 41438_2020_295_MOESM17_ESM.pdf]

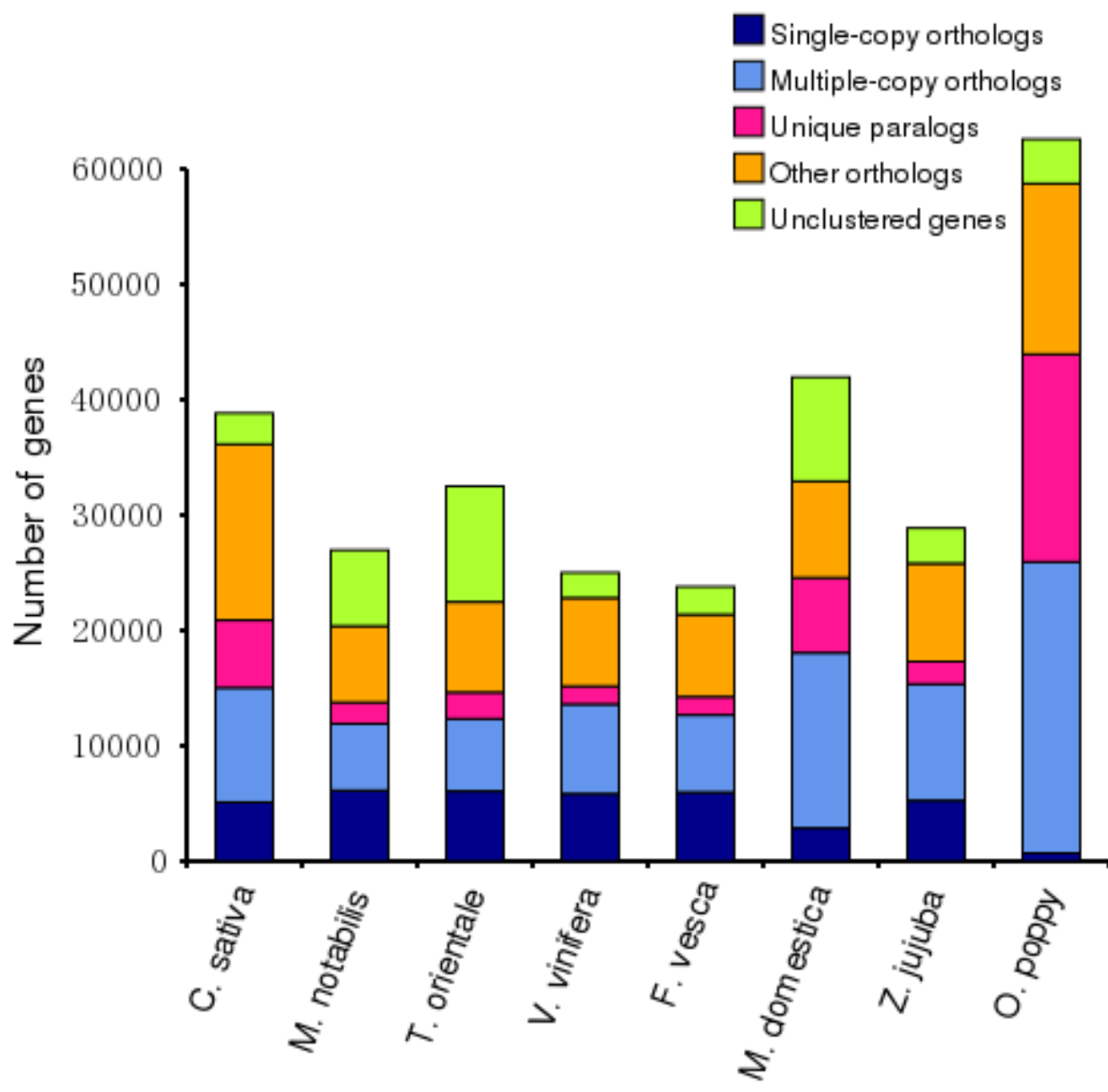

Supplement: Supplementary file 18 — Figure S5 [file 41438_2020_295_MOESM18_ESM.pdf]

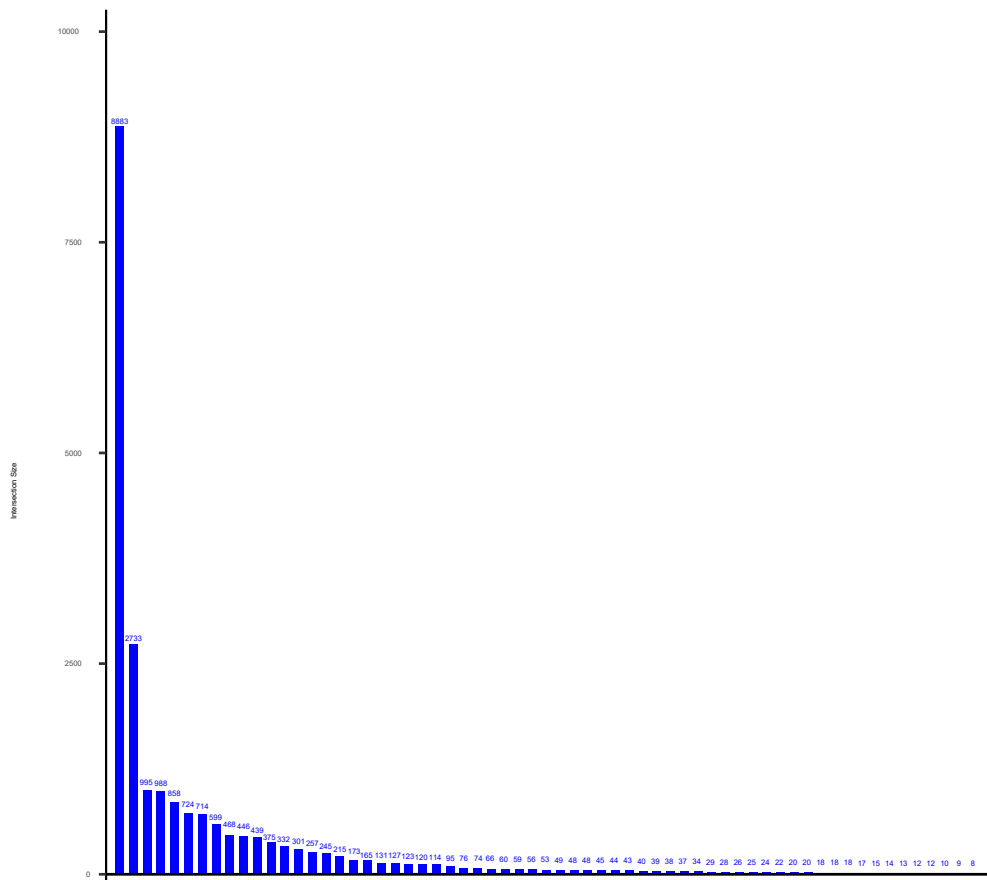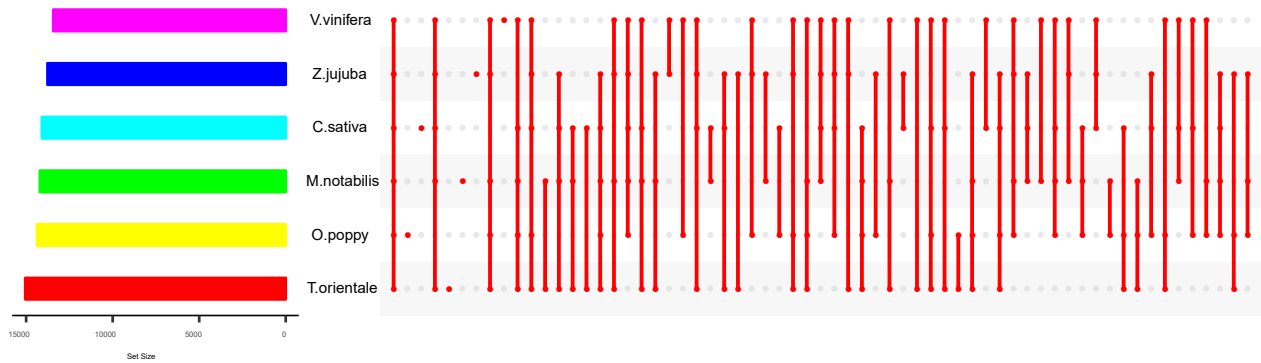

Supplement: Supplementary file 19 — Figure S6 [file 41438_2020_295_MOESM19_ESM.pdf]
